# Correction: SARS-CoV-2 antibody seroprevalence in Togo: a national cross-sectional household survey, May–June, 2021

**DOI:** 10.1186/s12889-023-15004-3

**Published:** 2023-01-27

**Authors:** Yao Rodion Konu, Siaka Condé, Fifonsi Gbeasor-Komlanvi, Arnold Junior Sadio, Martin Kouame Tchankoni, Joel Anani, Alexandra Bitty-Anderson, Bisimwa Ruhana Mirindi, Fatoumata Binta Tidiane Diallo, Moustapha MIjiyawa, Anoumou Claver Dagnra, Didier Koumavi Ekouevi

**Affiliations:** 1 Bordeaux Population Health Centre, UMR 1219, University of Bordeaux, National Institute for Health and Medical Research (INSERM), Research Institute for Sustainable Development (IRD), Bordeaux, France; 2 Département de Santé Publique, Université de Lomé, Lomé, Togo; 3 Centre de Recherche et de Formation en Santé Publique (CFRSP), Université de Lomé, Lomé, Togo; 4 Centre Africain de Recherche en Epidémiologie et en Santé Publique (CARESP), Lomé, Togo; 5 Togo Office, World Health Organization (WHO), Lomé, Togo; 6 Ministère de la Santé, de l’Hygiène Publique et de l’accès universel aux soins, Lomé, Togo; 7 Laboratoire de Biologie Moléculaire et d’Immunologie (BIOLIM), Université de Lomé, Lomé, Togo

**Correction: BMC Public Health 22, 2294 (2022)**

**https://doi.org/10.1186/s12889-022-14794-2**

The original publication [1] of this paper contained a typo in the abstract. The value "CI95%: 18.9–21.1" should have been "95%CI: 64.3–66.6". This was correct elsewhere in the article. The original article has been updated to correct this.
